# Model tests of dynamic response of modified lateritic soil embankments under combined action of wetting and vibration

**DOI:** 10.1038/s41598-025-03881-0

**Published:** 2025-05-29

**Authors:** Xue Han, Xili Qin, Hanhui He, Guang Yang, Jiale Gong

**Affiliations:** 1 College of Civil Engineering and Architecture, Hunan University of Arts and Science, Changde, 415000 China; 2 School of Architectural Engineering, Heilongjiang University of Science & Technology, Harbin, 150022 China

**Keywords:** Lateritic soil with high liquid limit, Embankment, Wetting, Dynamic response, Model test, Engineering, Civil engineering

## Abstract

Modifying lateritic soils, which are widely distributed in humid and rainy regions around the world, for embankment construction is a practical necessity for highway and railway projects. These embankments are susceptible to infiltration of rainfall, wetting and vibration from earthquakes and traffic. Further study is required to investigate the dynamic response characteristics of these embankments under combined action of wetting and vibration. Two scaled-down physical models of embankments were built: one with unmodified lateritic soils, which are typical soils with high liquid limit in central-southern China, and the other with lateritic soils modified with lime at a content of 8%. A self-designed model test system was used to conduct model tests of both embankments under combined action of wetting and vibration. White noise excitation was employed to quantitatively compare the two types of embankments in terms of variations of dynamic properties, such as natural frequency and damping ratio, with wetting degrees. Three types of seismic waves—Chi_Chi, NCALIF and SFERN—were used to quantitatively compare the two types of embankments in terms of variations of dynamic response parameters, including PGA amplification effect, pore water pressure and earth pressure, with wetting degrees and acceleration amplitudes. The test results reveal significant differences in dynamic properties and responses of the two types of embankments. Compared to the unmodified embankment, the damping ratio and PGA amplification factor of the modified embankment are reduced by up to 53.5% and 37.5%, respectively, resulting in an effective mitigation of the combined action of wetting and vibration. Test values of natural frequency, damping ratio, PGA amplification factor, dynamic pore water pressure and dynamic earth pressure of both types of embankments are presented. The research findings provide a theoretical basis for highway and railway construction and for revision of technical specifications in regions with widespread lateritic soils.

## Introduction

Lateritic soils are widely distributed around the world, covering an area of 1.08 million square kilometers in China alone, which accounts for approximately 11.3% of the country’s land area^1,2^. Due to their high liquid limit, difficulty in compaction, and typical moisture sensitivity, lateritic soils are generally not suitable for direct use in embankment construction^3^. However, in highway and railway construction projects where lateritic soils are extensively distributed, discarding the local lateritic soils and importing soil from other locations for embankment construction would require new landfills and borrow pits. This is neither environmentally friendly nor economical in the context of increasingly stringent environmental protection requirements and limited land availability^4^. Therefore, It is an intrinsic requirement of highway and railway construction to modify the local lateritic soils and use them for embankment construction.

Existing research on the use of lateritic soils in highway construction has focused on the improvement of engineering properties, including attempts to modify lateritic soils with various materials such as lime and to explore the modification approaches. The practical demand for improving the engineering properties of lateritic soils for highway use has driven the theoretical research forward. These studies have examined the macroscopic physical characteristics, microscopic structural features, modification mechanisms, and physical and mechanical properties of lateritic soils modified with different materials and proportions from a material perspective^5–12^. Additionally, some researchers have studied the effects of contents of modifying materials on compaction characteristics and settlement of embankment from an engineering perspective^13–21^. In addition to commonly used modification materials such as lime and cement, there have been attempts to modify lateritic soils with other materials such as fly ash geopolymers and calcium carbide slag^22–36^. The studies and engineering practices have indicated that modification of lateritic soils with lime is both cost-effective and practical, and is still a subject worthy of further study.

The wetting effect and engineering challenges of modified lateritic soils, which are often used in regions with high rainfall and humidity, have increasingly drawn academic attention in recent years^37–39^. Field tests have been conducted on the dynamic response and characteristics of lateritic soil subgrade under combined action of wetting and cyclic loading^40^. Embankments constructed with modified lateritic soils are often not only subject to wetting because of rainwater infiltration and soaking but also to vibration from earthquakes and vehicles. Therefore, further study is required to investigate the dynamic response of these embankments under combined action of wetting and vibration. Two scaled down physical models of embankments were built, one with unmodified lateritic soils which are typical soils with high liquid limit from central-southern China, and the other one with lateritic soils modified with lime at a content of 8%. Using a self-designed test system, model tests were conducted on both embankments to investigate the dynamic responses under combined action of wetting and vibration. The purpose of this study is to provide a theoretical basis for construction of highways and railways and for revision of technical standards in regions where lateritic soils are extensively distributed.

## Design of model test

### Model test system and test materials

#### Introduction to the model test system

The main components of the model test system include a model box, a vibration table, a vibration loading system, an industrial control computer with loading control software, a controllable rainfall system and a high-frequency dynamic data acquisition and analysis system^41^.

The dimensions of the model box are 2.0 m × 1.5 m × 1.7 m. The maximum load of the vibration table is 9 tons while the allowable weight of the model is 6 tons. The frequency range of the vibration loading system is 0–50 Hz; the maximum acceleration under full load is 1 *g*; and the maximum displacement of vibration is ± 150 mm. The rainfall intensity, duration and volume of the controllable rainfall system can be adjusted by the flow meter and controllers.

#### Materials of the model and the properties

Natural lateritic soils with high liquid limit from central-southern China were used to build the physical models of the embankments. Two embankment models were built, one with unmodified lateritic soils and the other with lateritic soils modified with lime. Calcitic quicklime was used for the modification, with an effective content of (CaO + MgO) ≥ 75% and an content of MgO ≤ 5%. Based on the physical properties of the lateritic soils used in the test, the existing engineering experience on the modification of lateritic soils for road use and Reference^42^, the lime content was determined to be 8% by mass. When modified with lime, the liquid limit of the lateritic soil was reduced to below 50%, and the plastic limit was increased to above 30%, which met the technical requirements of fill materials for embankment. The optimal moisture content for compaction of the modified lateritic soil was found to be 19–20%. The major parameters of the natural and modified lateritic soils, as determined by geotechnical test, are presented in Table 1.

**Table 1:** Major parameters of the natural and modified lateritic soils.

Material	Liquid limit (%)	Plastic limit (%)	Plasticity index I_*P*_	Optimal moisture content for compaction (%)	Maximum dry density (g/cm^3^)
Natural lateritic soil	53.2	26.3	26.9	18	1.57
Lateritic soil modified with lime at a content of 8%	46.7	35.8	10.9	19–20	1.52

### Design and construction of the physical models of embankments

#### Similarity design of the model

Dimensional analysis was used for the similarity design of the scaled physical model based on Buckingham’s π theorem. The similarity constants of the embankment model were calculated and determined by considering the stress-strain relationships of the prototype embankment and the model, as well as the geometric dimensions and boundary conditions of the model box.

The ratio of geometric similarity between the prototype and the model is *C*_L_=*L*_P_/*L*_m_=15, where *L*_P_ is the dimension of the prototype and *L*_m_ is the dimension of the model. The similarity constants of variables of the physical models are presented in Table 2.

**Table 2:** Similarity constants of embankment models built with lateritic soils.

Variable	Similarity ratio	Similarity constant	Description
Geometric dimension *L*	*C*_*L*_	15	Fundamental variable
Seismic acceleration *a*	*C*_*a*_	1	Prototype material
Density *ρ*	*C*_*ρ*_	1	Prototype material
Internal friction angle *φ*	*C*_*φ*_	1	Prototype material
Poisson’s ratio *μ*	*C*_*μ*_	1	Prototype material
Gravitational acceleration *g*	*C*_*g*_=*C*_*a*_	1	Prototype material
Frequency *ω*	${C_\omega }=C_{a}^{{1/2}}/C_{L}^{{1/2}}$	0.26	–
Shear modulus *G*	${C_G}={C_L}{C_\rho }{C_a}$	15	–
Cohesion *c*	${C_c}={C_L}{C_\rho }{C_a}$	15	–
Dimensionless coefficient *K*	*C*_*K*_	1	Prototype material
Stress *σ*	${C_\sigma }={C_L}{C_\rho }{C_a}$	15	–
Strain *ε*	*C*_*ε*_=1	1	Prototype material
Displacement *S*	*C*_*s*_=*C*_*L*_	15	Similarity constant was taken into account in data processing
Time *t*	${C_t}=C_{L}^{{1/2}}/C_{a}^{{1/2}}$	3.87	Time of seismic wave input was scaled according to the similarity constant
Velocity *V*	${C_V} - C_{L}^{{1/2}}C_{a}^{{1/2}}$	3.87	Similarity constant was taken into account in data processing

#### Construction of the physical models and layout of sensors

Based on the dimensions of the prototype and the geometric similarity constants of the physical models, dimensions of the cross section of the models were calculated and determined as follows: the total height was 900 mm with a slope height of 600 mm and a base thickness of 300 mm; width of the base was 1725 mm and gradient of the slope was 1:1.25.

The unmodified lateritic soil for the embankment model was prepared as follows. Lateritic soil excavated from the field was first air-dried in the laboratory until the moisture content was less than 18%. The soil was then crushed, rolled and sieved to remove the large clumps. Before the soil was used to build the model, the moisture content of the soil was measured and the required amount of water was calculated. Then the required water was sprayed to the soil to adjust the moisture content to the optimal 18% for compaction.

The modified lateritic soil for the embankment model was prepared as follows. Lateritic soil excavated from the field was first air-dried in the laboratory until the moisture content was around 20%. The soil was then crushed, rolled and sieved to remove the large clumps. Lime was added to the soil at a mass ratio of 8% and thoroughly mixed with the soil. Before the soil-lime mixture was used to build the model, the moisture content of the mixture was measured and the required amount of water was calculated and sprayed to the mixture to adjust the moisture content to the optimal 20% for compaction.

Before the construction of the embankment model, the interface between the embankment model and the model box was treated to minimize boundary effects on the test results. A layer of fine crushed stones with a maximum particle size of 2 mm was laid at the bottom of the model box to prevent relative sliding between the embankment soil and the box during vibration. Additionally, in order to mitigate interference from the reflected waves during vibration, a board of polyethylene foam with a thickness of 50 mm was placed on the inner side of the model box, perpendicular to the direction of vibration.

The physical model was built in nine layers, each with a thickness of 100 mm, and the compaction degree was 95%. The weight of soil for each layer was calculated based on the compaction degree and the thickness of the layer. Then the prepared soil was weighed and evenly spread in the model box. A straightedge was used to scrape and level the surface to ensure uniform thickness of the layer. Then a square board of hardwood was placed on the surface of the soil, and the soil is compacted by hammering the board until the thickness of the layer reached 100 mm. The surface of the layer was then roughened before the next layer was spread and compacted in the same manner. This process proceeded layer by layer. When the embankment model was completed, a plastic film was placed over the model and sealed to prevent the surface from drying. The model was then left undisturbed for a week to allow the interfaces between the layers to bond and thus to enhance the integrity of the model^43^.

During the construction of the physical model, sensors were embedded in the embankment as required for test. Five accelerometers (A1–A5) were placed at different heights along the slope surface. At the toe of the slope, three dynamic earth pressure transducers (P1–P3) were installed at the same level, and three dynamic pore water pressure transducers (F1–F3) were installed at the same level. Additionally, one accelerometer (A6) was fixed on the shaking table surface outside the embankment model.

**Fig. 1:**
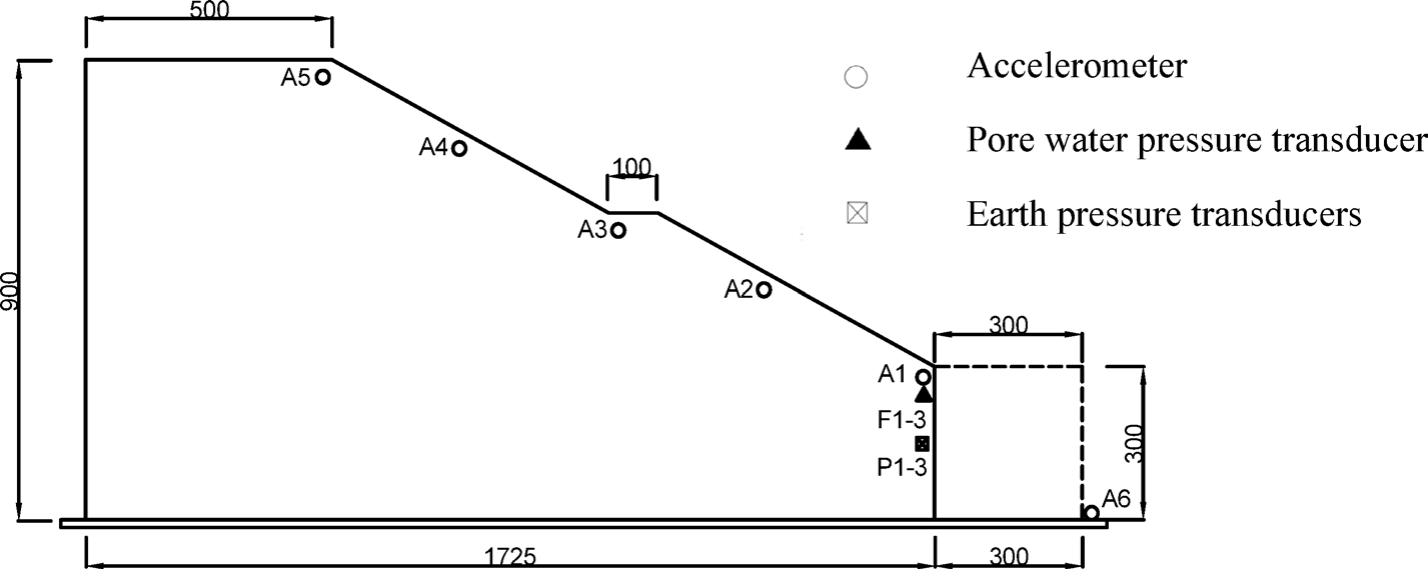
Cross section of the embankment model and layout of sensors.

**Fig. 2:**
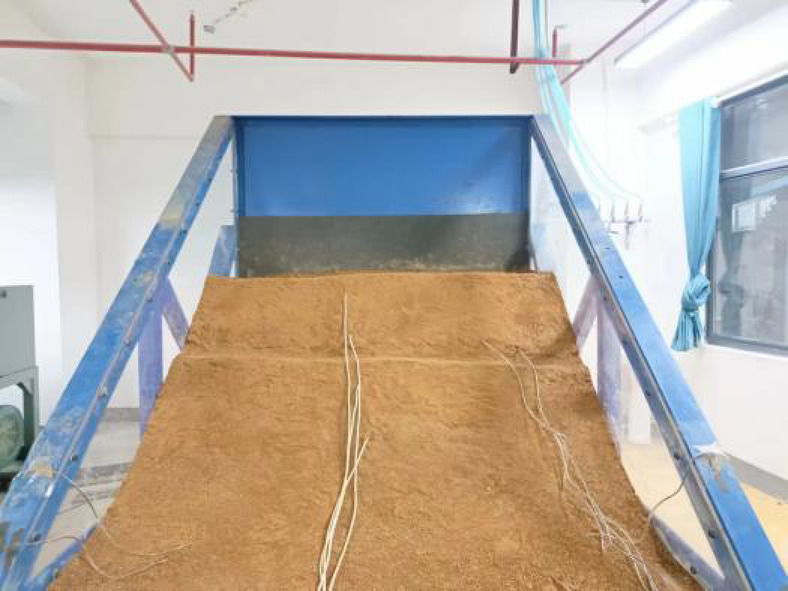
The physical model of embankment constructed with unmodified lateritic soil.

**Fig. 3:**
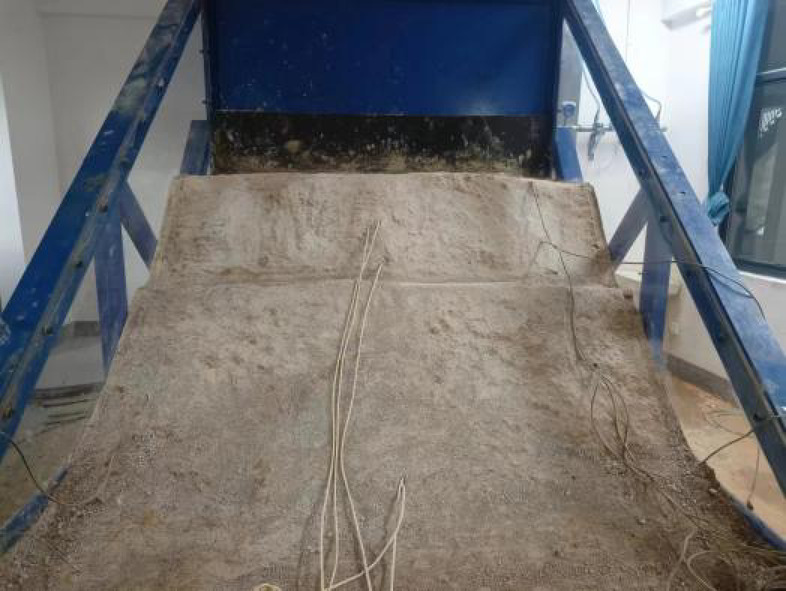
The physical model of embankment constructed with modified lateritic soil.

Both physical models were constructed by the same approaches and standards to ensure consistency between the models. The cross-sectional dimensions of the physical models and the layout of the sensors are shown in Fig. 1. Figures 2 and 3 present the actual photographs of the completed physical models of the embankments constructed with unmodified and modified lateritic soils, respectively.

### Data acquisition

The data acquisition system used in the test was an INV3065N2 high-frequency multi-channel dynamic data acquisition instrument, which was connected to a computer via data cables and was equipped with the DASP-V11 professional data acquisition software. Dynamic response data from sensors, such as acceleration, pore water pressure and earth pressure, were displayed in real-time and stored in the computer through the acquisition instrument and software. Additionally, the collected data were filtered and baseline corrected.

### Dynamic properties test of the embankments

White noise was used in the dynamic properties test to excite both the unmodified and modified lateritic soil embankments. The acceleration response was measured, and the natural frequency and damping ratio of the embankments were calculated. The relationships between these dynamic properties and the wetting degrees were compared and analyzed.

### Scheme of white noise excitation

The unmodified and modified lateritic soil embankment models were subjected to controlled rainfall and infiltration to achieve different degrees of wetting, which were defined by the percentages of wetted volume to the total volume of the embankment. A rainfall spray system was used for pressurized and atomized spraying. The amount and duration of atomized rainfall can be regulated to achieve uniform infiltration and to control the wetting levels. Seven wetting degrees were achieved, ranging from 0% (no wetting) to 48%, with an increment of 8%.

**Fig. 4:**
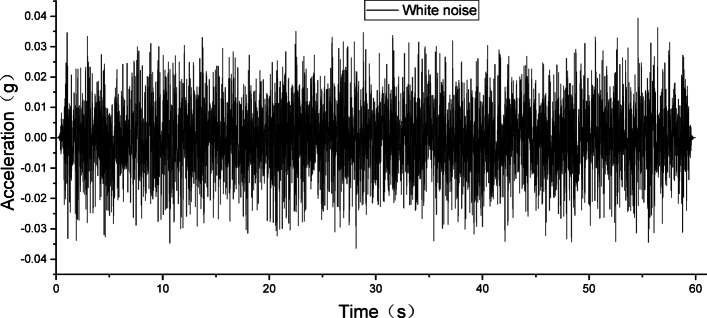
Acceleration time history of white noise.

At different wetting levels, the models were excited with white noise for 60 s, with a peak acceleration of 0.035 *g*. Figure 4 shows the acceleration time history of the applied white noise excitation.

### Analysis of results of dynamic properties test

To compare and analyze the differences in dynamic properties between modified and unmodified lateritic soil embankments, curves were plotted to show the relationships between natural frequency and wetting degree, as well as the relationships between damping ratio and wetting degree.

**Fig. 5:**
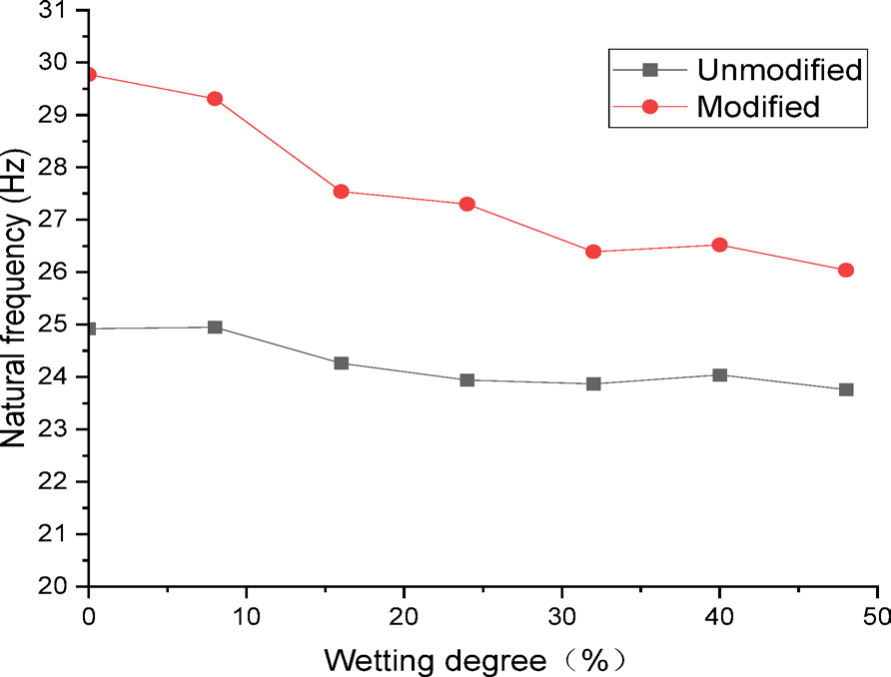
Curve of natural frequency versus wetting degree.

Figure 5 presents the curves of natural frequency of the embankments versus wetting degree. Analysis of the curves indicates that, at the same wetting degree, the natural frequency of the modified lateritic soil embankment is significantly greater than that of the unmodified embankment. At a wetting degree of 0%, the natural frequency of the modified lateritic soil embankment is 29.77 Hz, which is 1.195 times that of the unmodified lateritic soil embankment at 24.92 Hz; at a wetting degree of 48%, the natural frequency of the modified embankment is 26.04 Hz, which is 1.096 times that of the unmodified embankment at 23.76 Hz. The natural frequencies of both embankments decrease as the wetting degree increases. The rate of decrease in natural frequency of the modified lateritic soil embankment is obviously greater than that of the unmodified embankment.

**Fig. 6:**
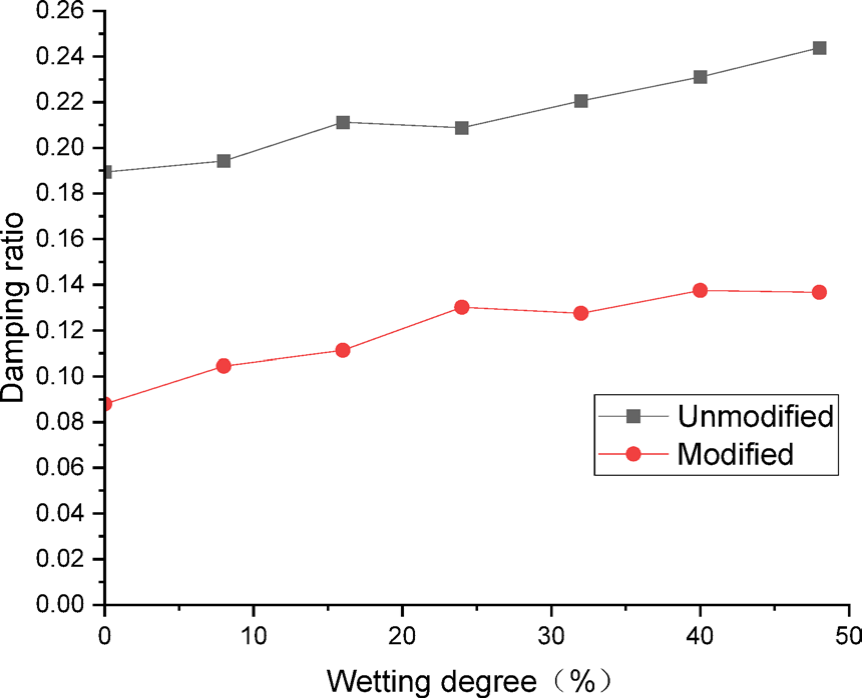
Curve of damping ratio versus wetting degree.

Figure 6 presents the curves of damping ratio of the embankments versus wetting degree. Analysis of the curves indicates that, at the same wetting degree, the damping ratio of the modified lateritic soil embankment is significantly lower than that of the unmodified embankment. At a wetting degree of 0%, the damping ratio of the unmodified lateritic soil embankment is 0.1894, which is 2.15 times that of the modified lateritic soil embankment at 0.088; at a wetting degree of 48%, the damping ratio of the unmodified lateritic soil embankment is 0.2438, which is 1.78 times that of the modified lateritic soil embankment at 0.1367. The damping ratios of both embankments increase as the wetting degree increases. The rate of increase in damping ratio of the unmodified lateritic soil embankment is approximately equal to that of the modified embankment. Compared to the unmodified embankment, the damping ratio of the modified embankment is reduced by up to 53.5%.

## Dynamic response test of the embankment

### Loading scheme of the dynamic response test

The embankment models were subjected to combined action of wetting and vibration. Through simulated rainfall, the embankment models were wetted to three levels, which were defined by the percentages of wetted volume to the total volume of the embankment: 0% (no wetting), 10% and 40%, and three types of seismic waves were applied to measure the dynamic response parameters of the embankment.

**Fig. 7:**
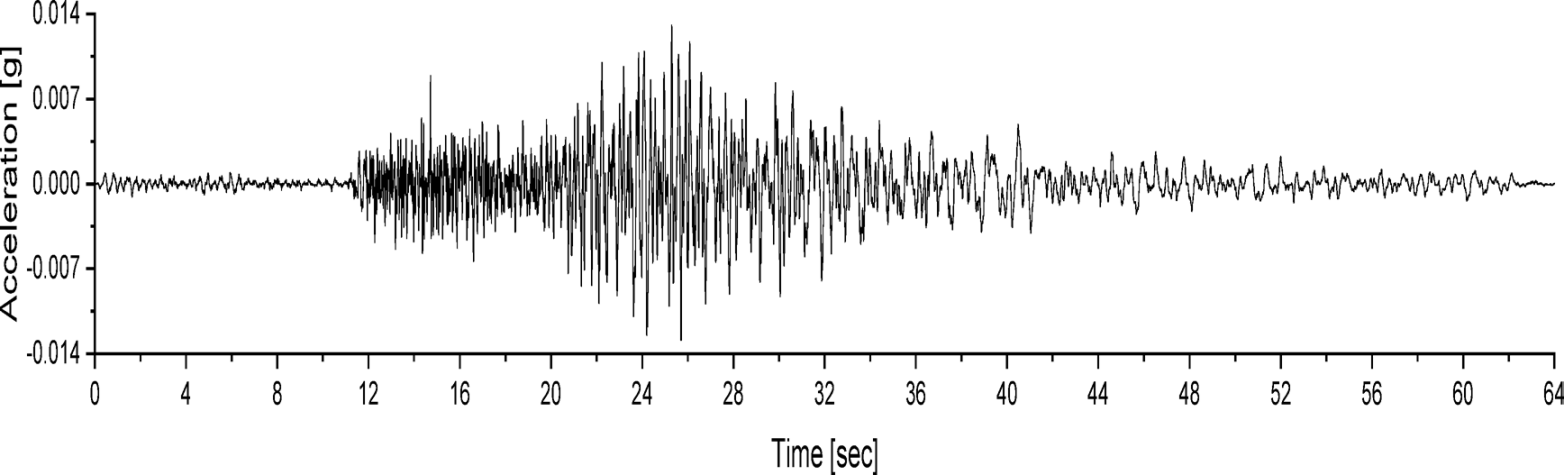
Acceleration time history of the Chi_Chi wave.

**Fig. 8:**
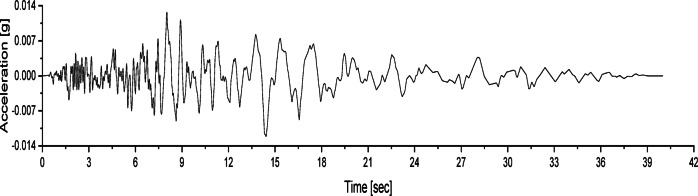
Acceleration time history of the NCALIF wave.

**Fig. 9:**
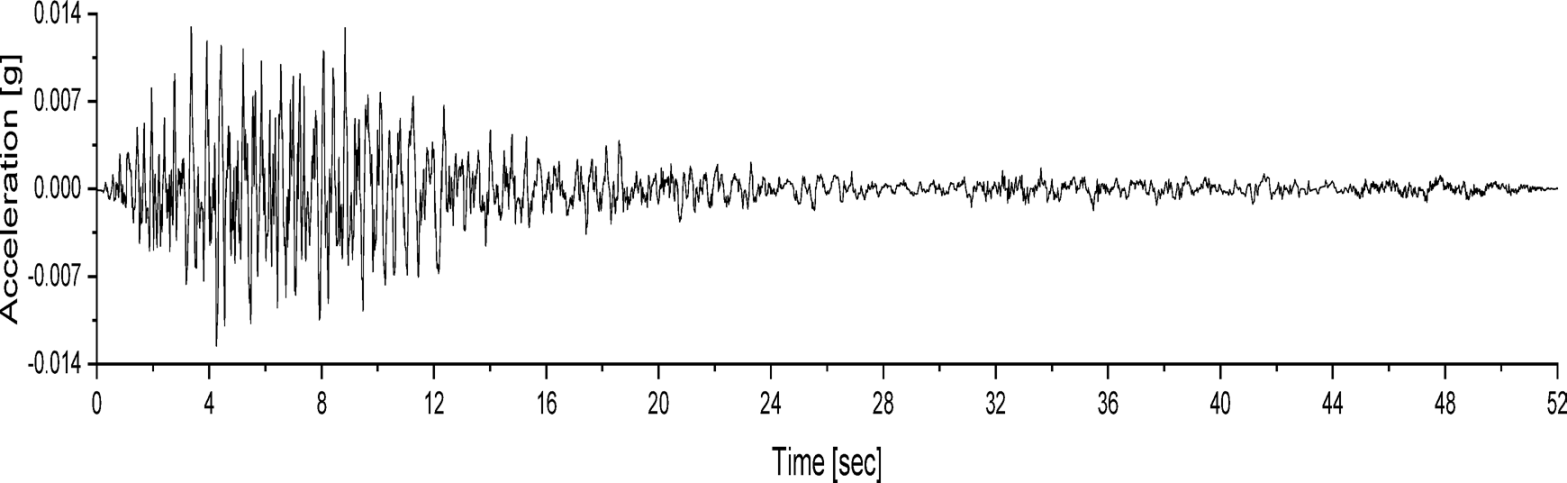
Acceleration time history of the SFERN wave.

Three types of seismic waves, namely the Chi_Chi, NCALIF and SFERN, were applied for the vibration test of the models. These seismic waves were used as base waves and were scaled according to the similarity constants of the model tests. The acceleration time histories of the three base waves are shown in Figs. 7, 8 and 9, and the loading scheme of the seismic waves is detailed in Table 3. During the tests, the acceleration amplitudes were scaled to achieve different levels of seismic intensity.

**Table 3:** Loading scheme for the vibration of embankment models.

No.	Type of seismic wave	Acceleration amplitude of the seismic wave (g)	No.	Type of seismic wave	Acceleration amplitude of the seismic wave (g)
1	Chi_Chi	0.01	16	Chi_Chi	0.20
2	NCALIF	0.01	17	NCALIF	0.20
3	SFERN	0.01	18	SFERN	0.20
4	Chi_Chi	0.03	19	Chi_Chi	0.25
5	NCALIF	0.03	20	NCALIF	0.25
6	SFERN	0.03	21	SFERN	0.25
7	Chi_Chi	0.05	22	Chi_Chi	0.30
8	NCALIF	0.05	23	NCALIF	0.30
9	SFERN	0.05	24	SFERN	0.30
10	Chi_Chi	0.10	25	Chi_Chi	0.35
11	NCALIF	0.10	26	NCALIF	0.35
12	SFERN	0.10	27	SFERN	0.35
13	Chi_Chi	0.15	28	Chi_Chi	0.40
14	NCALIF	0.15	29	NCALIF	0.40
15	SFERN	0.15	30	SFERN	0.40

### Analysis of the results of dynamic response test

Acceleration, dynamic pore water pressure, and dynamic earth pressure at instrumented points in the unmodified and modified lateritic soil embankment models were measured under combined action of wetting and vibration. A comparative analysis is conducted on the acceleration amplification effect, peak dynamic pore water pressure, and peak dynamic earth pressure.

#### Analysis of acceleration amplification effect

The PGA (Peak Ground Acceleration) amplification factor, which quantifies the amplification effect of acceleration in the embankment, was calculated for different points (A1, A2, A3, A4 and A5) on the slope of the embankments. The average value of the five points was subsequently calculated to represent the global PGA amplification factor of the embankment.

**Fig. 10:**
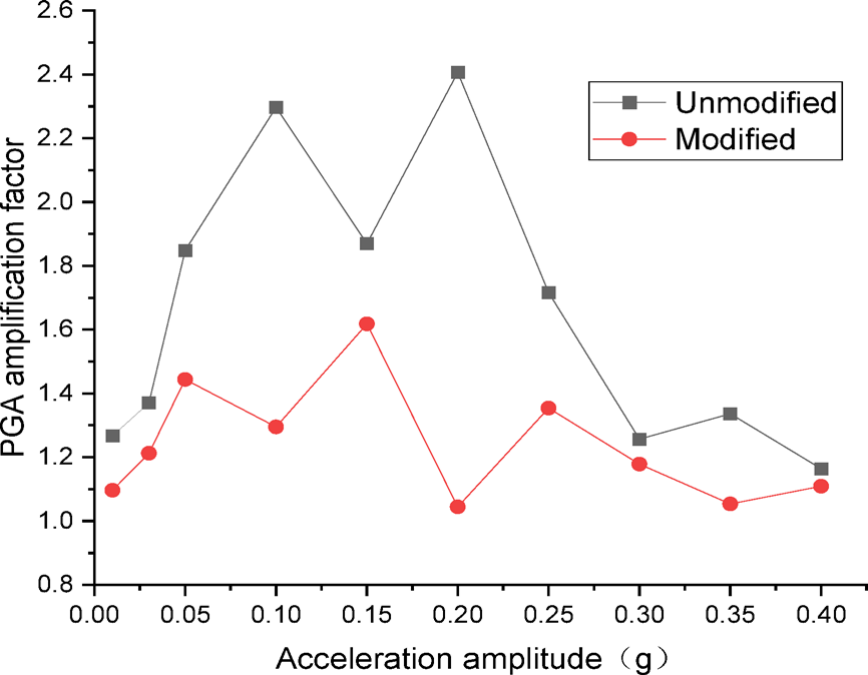
PGA amplification factor under Chi_Chi wave excitation at a wetting degree of 0%.

**Fig. 11:**
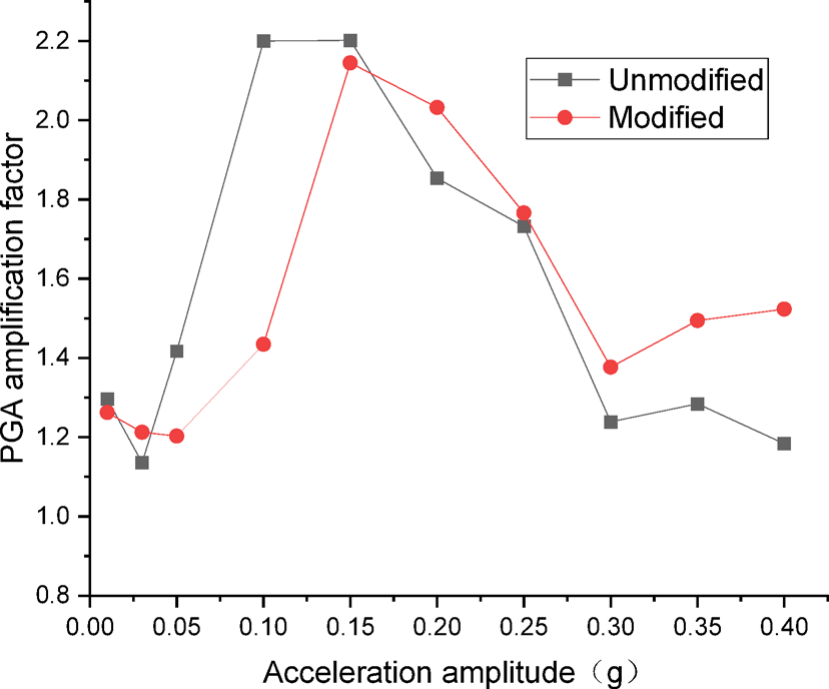
PGA amplification factor under Chi_Chi wave excitation at a wetting degree of 40%.

Figures 10 and 11 present the curves of PGA amplification factor versus acceleration amplitude for the embankment under Chi_Chi wave excitation. As illustrated in Fig. 10 for the wetting degree of 0%, the maximum PGA amplification factor of the unmodified lateritic soil embankment is 2.41, occurring at the acceleration amplitude of 0.20 *g*, while the maximum PGA amplification factor of the modified lateritic soil embankment is 1.62, occurring at the acceleration amplitude of 0.15 *g*. As shown in Fig. 11 for the wetting degree of 40%, the maximum PGA amplification factor of the unmodified embankment is 2.20 and that of the modified embankment is 2.14, both of which occur at the acceleration amplitude of 0.15 *g*.

**Fig. 12:**
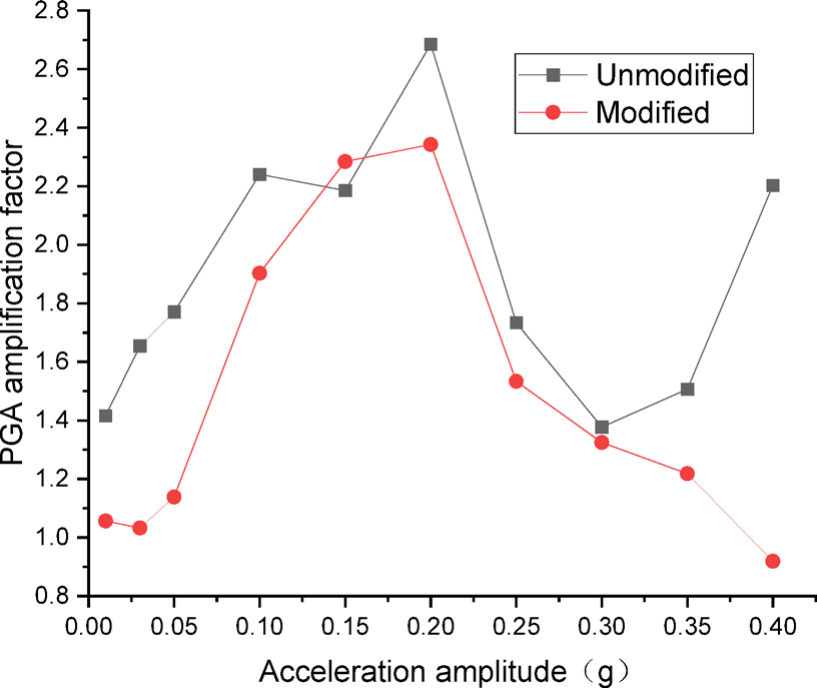
PGA amplification factor under NCALIF wave excitation at a wetting degree of 0%.

**Fig. 13:**
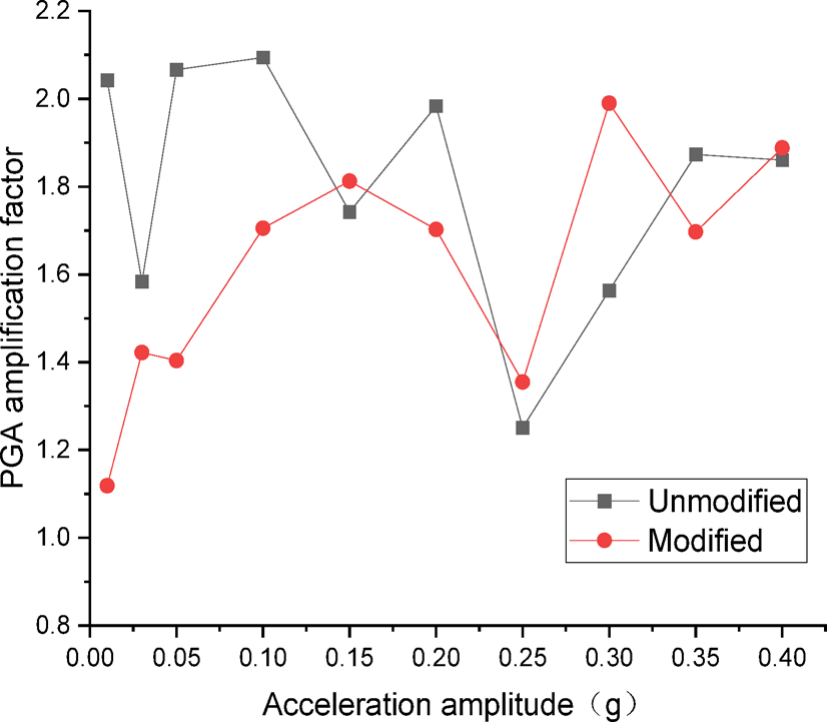
PGA amplification factor under NCALIF wave excitation at a wetting degree of 40%.

Figures 12 and 13 present the curves of PGA amplification factor versus acceleration amplitude for the embankment under NCALIF wave excitation. As shown in Fig. 12 for the wetting degree of 0%, the maximum PGA amplification factor of the unmodified embankment is 2.69 and that of the modified embankment is 2.34, both of which at the acceleration amplitude of 0.20 *g*. As illustrated in Fig. 13 for the wetting degree of 40%, the maximum PGA amplification factor of the unmodified lateritic soil embankment is 2.09, occurring at the acceleration amplitude of 0.10 *g*, while the maximum PGA amplification factor of the modified lateritic soil embankment is 1.99, occurring at the acceleration amplitude of 0.30 *g*.

**Fig. 14:**
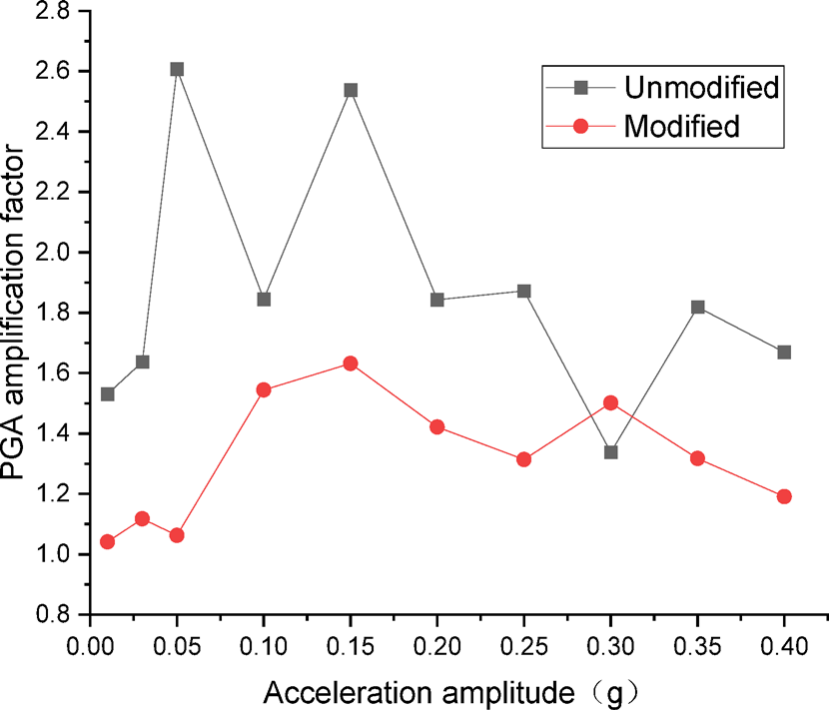
PGA amplification factor under SFERN wave excitation at a wetting degree of 0%.

**Fig. 15:**
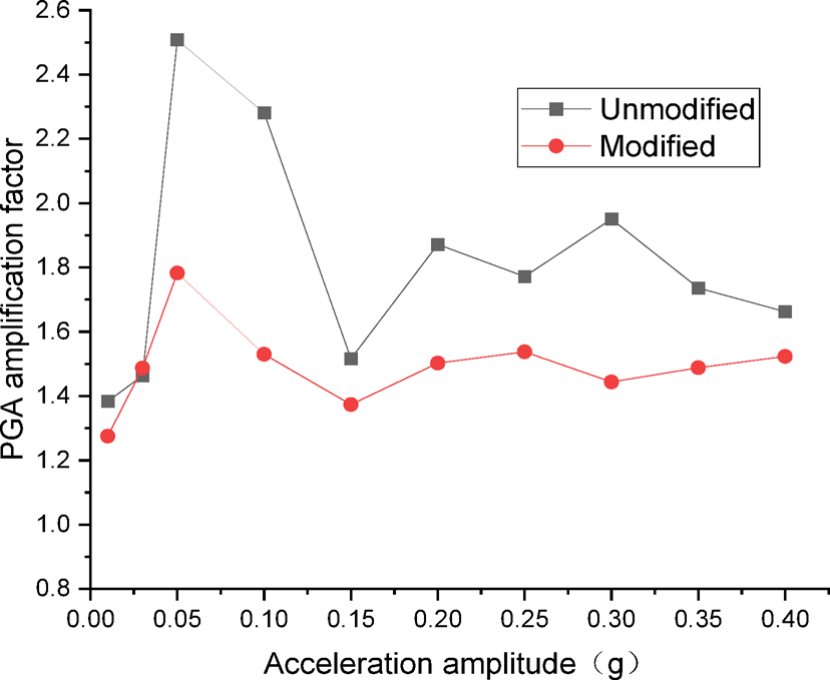
PGA amplification factor under SFERN wave excitation at a wetting degree of 40%.

Figures 14 and 15 present the curves of PGA amplification factor versus acceleration amplitude for the embankment under SFERN wave excitation. As illustrated in Fig. 14 for the wetting degree of 0%, the maximum PGA amplification factor of the unmodified lateritic soil embankment is 2.61, occurring at the acceleration amplitude of 0.05 *g*, while the maximum PGA amplification factor of the modified lateritic soil embankment is 1.63, occurring at the acceleration amplitude of 0.15 *g*. As shown in Fig. 15 for the wetting degree of 40%, the maximum PGA amplification factor of the unmodified embankment is 2.51 and that of the modified embankment is 1.78, both of which occur at the acceleration amplitude of 0.05 *g*.

A comparison of the PGA amplification effect under the action of the three types of seismic waves indicates that, at the same wetting degree, the maximum PGA amplification factor of the modified embankment is consistently lower than that of the unmodified embankment. This suggests that modifying the lateritic soil for embankment construction reduces the PGA amplification effect of the embankment, thereby achieving better performance in seismic mitigation. The maximum PGA amplification factors for both types of embankments predominantly occur within the low acceleration amplitude range of no more than 0.20 *g*. Compared to the unmodified embankment, the PGA amplification factor of the modified embankment under the excitation of SFERN wave is reduced by up to 37.5%. Under the same working conditions, the stiffness of the modified embankment is increased when compared to the unmodified embankment. The increased stiffness reduces the transmission of low-frequency vibrations such as those from earthquakes. As a result, the reduction of PGA amplification factor effectively reduces the intensity of seismic forces actually experienced by the embankment and thereby mitigates the destructive effects of seismic inertial forces.

#### Analysis of dynamic pore water pressure

**Fig. 16:**
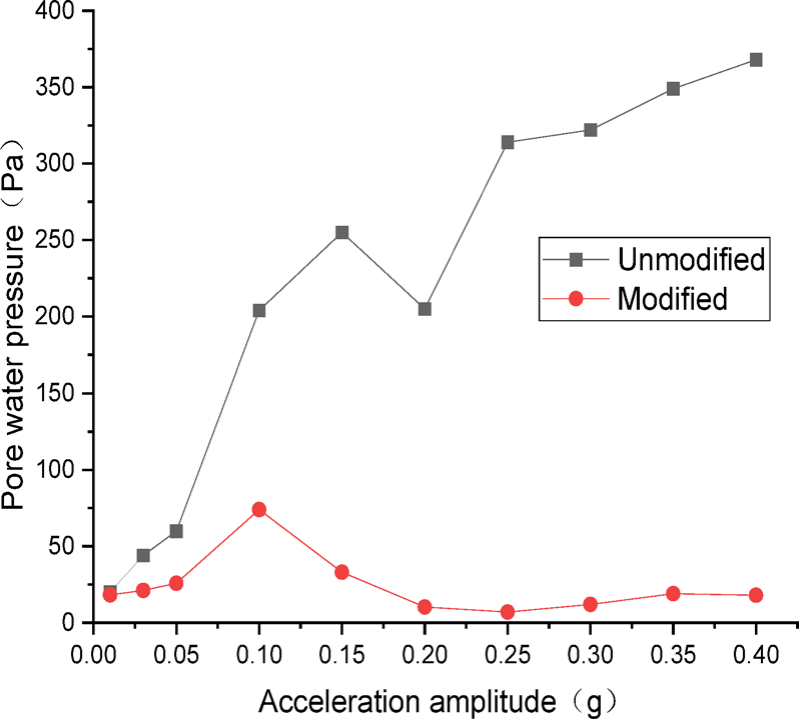
Pore water pressure under Chi_Chi wave excitation at a wetting degree of 10%.

**Fig. 17:**
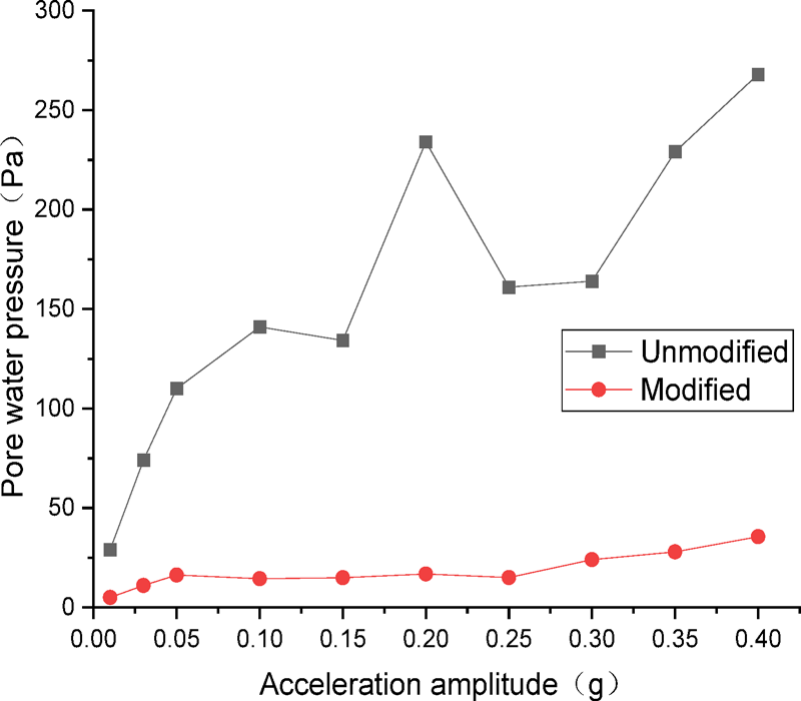
Pore water pressure under Chi_Chi wave excitation at a wetting degree of 40%.

The data from dynamic pore water pressure transducers were processed and plotted as curves. Figures 16 and 17 show the curves of peak dynamic pore water pressure versus acceleration amplitude for the embankment under Chi_Chi wave excitation. As indicated in Fig. 16 for the wetting degree of 10%, the maximum dynamic pore water pressure of the unmodified lateritic soil embankment is 368 Pa, which is 4.97 times that of the modified embankment at 74 Pa. As indicated in Fig. 17 for the wetting degree of 40%, the maximum dynamic pore water pressure of the unmodified lateritic soil embankment is 268 Pa, which is 7.4 times that of the modified embankment at 36 Pa.

**Fig. 18:**
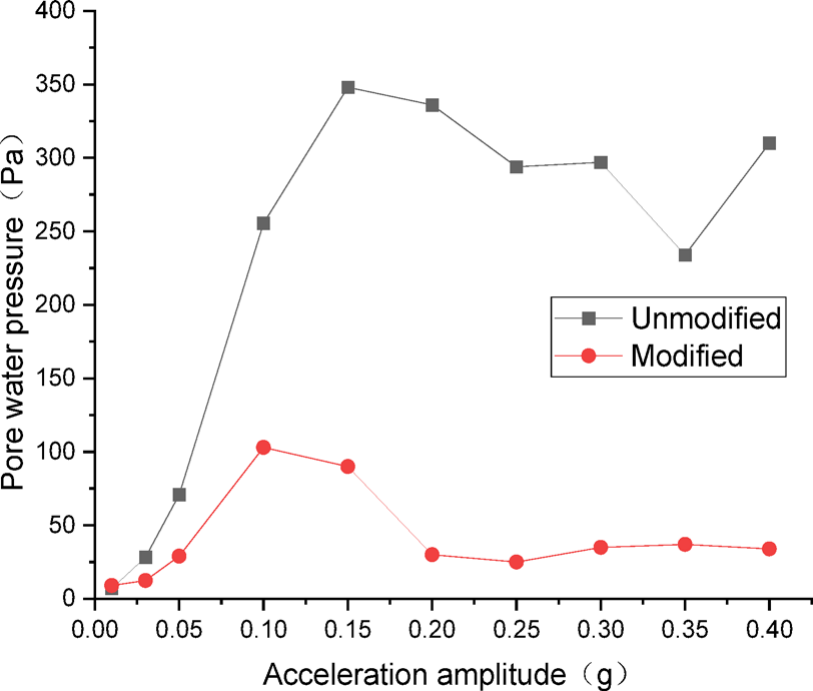
Pore water pressure under NCALIF wave excitation at a wetting degree of 10%.

**Fig. 19:**
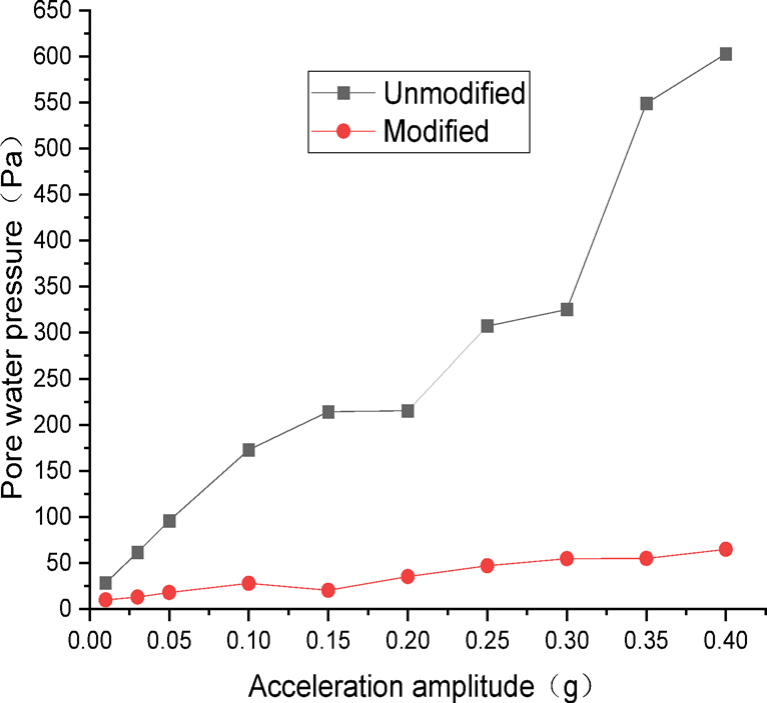
Pore water pressure under NCALIF wave excitation at a wetting degree of 40%.

Figures 18 and 19 show the curves of peak dynamic pore water pressure versus acceleration amplitude for the embankment under NCALIF wave excitation. As indicated in Fig. 18 for the wetting degree of 10%, the maximum dynamic pore water pressure of the unmodified lateritic soil embankment is 348 Pa, which is 3.38 times that of the modified embankment at 103 Pa. As indicated in Fig. 19 for the wetting degree of 40%, the maximum dynamic pore water pressure of the unmodified lateritic soil embankment is 603 Pa, which is 9.28 times that of the modified embankment at 65 Pa.

**Fig. 20:**
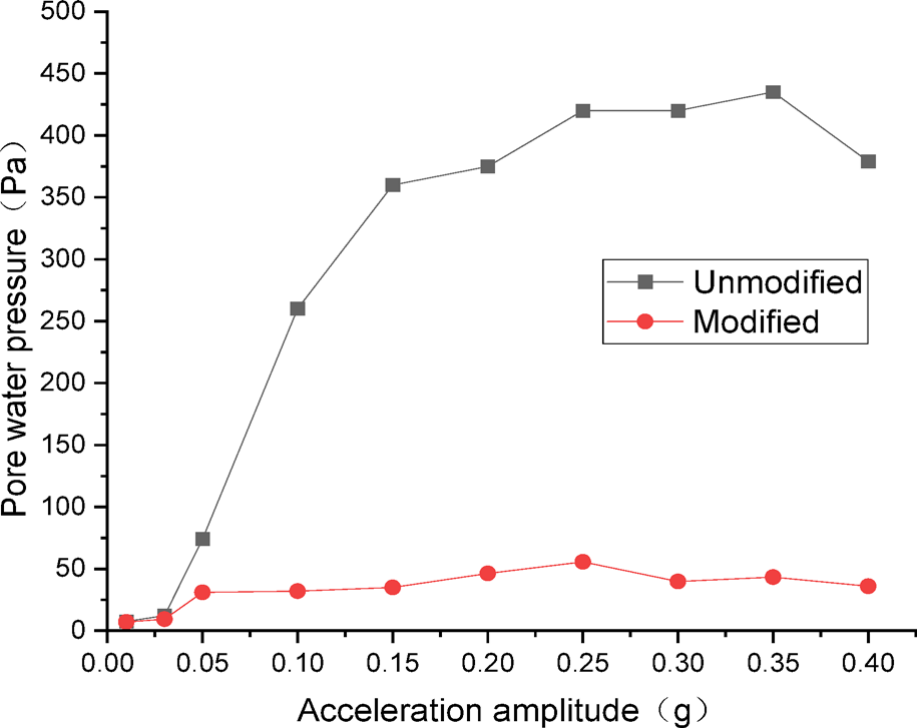
Pore water pressure under SFERN wave excitation at a wetting degree of 10%.

**Fig. 21:**
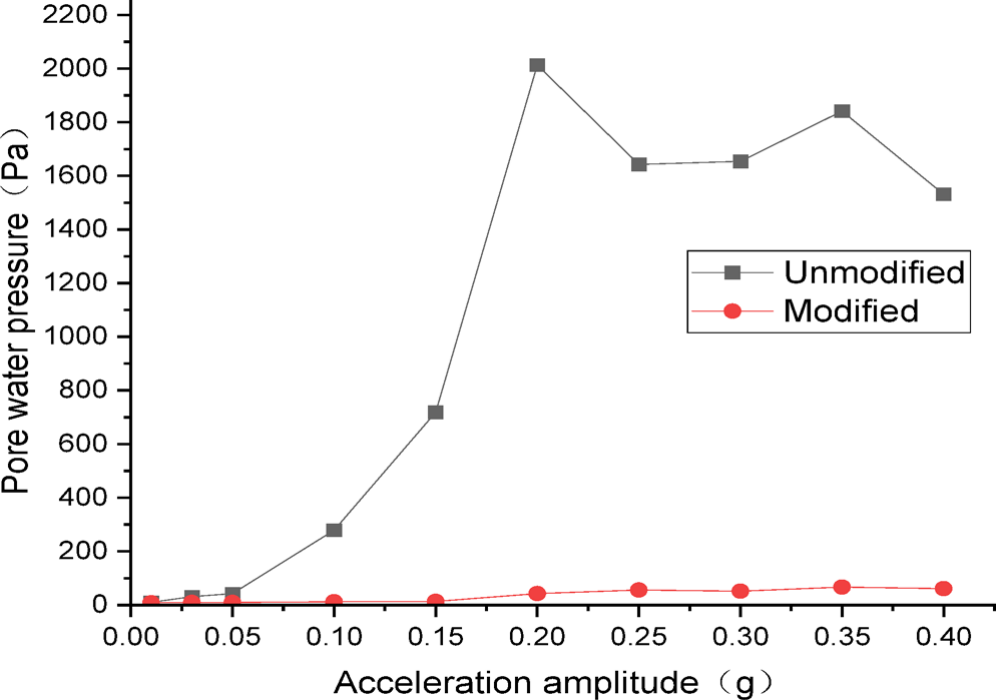
Pore water pressure under SFERN wave excitation at a wetting degree of 40%.

Figures 20 and 21 show the curves of peak dynamic pore water pressure versus acceleration amplitude for the embankment under SFERN wave excitation. As indicated in Fig. 20 for the wetting degree of 10%, the maximum dynamic pore water pressure of the unmodified lateritic soil embankment is 435 Pa, which is 7.82 times that of the modified embankment at 55.6 Pa. As indicated in Fig. 21 for the wetting degree of 40%, the maximum dynamic pore water pressure of the unmodified lateritic soil embankment is 2013 Pa, which is 30.04 times that of the modified embankment at 67 Pa.

Analysis of the curves of the three types of seismic waves indicates that, the peak dynamic pore water pressure of the modified lateritic soil embankment is significantly lower than that of the unmodified embankment whether in the state of low wetting degree of 10% or high wetting degree of 40%. This indicates that the modification of lateritic soil with lime greatly improves the soil’s permeability, resulting in a dissipation of the pore water pressure. Therefore, the dynamic pore water pressure in the soil is effectively reduced. Compared to the unmodified embankment, the lower pore water pressure in the modified embankment under seismic action reduces the total stress experienced by the soil, increases the effective shear strength of the soil, and thereby improves the seismic performance of the embankment.

#### Analysis of dynamic Earth pressure

**Fig. 22:**
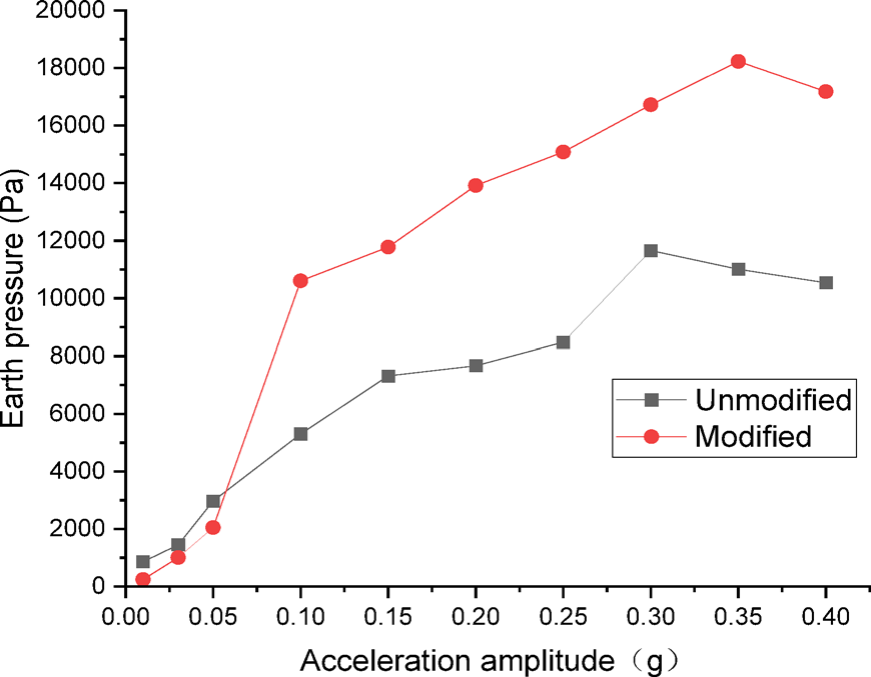
Earth pressure under Chi_Chi wave excitation at a wetting degree of 0%.

**Fig. 23:**
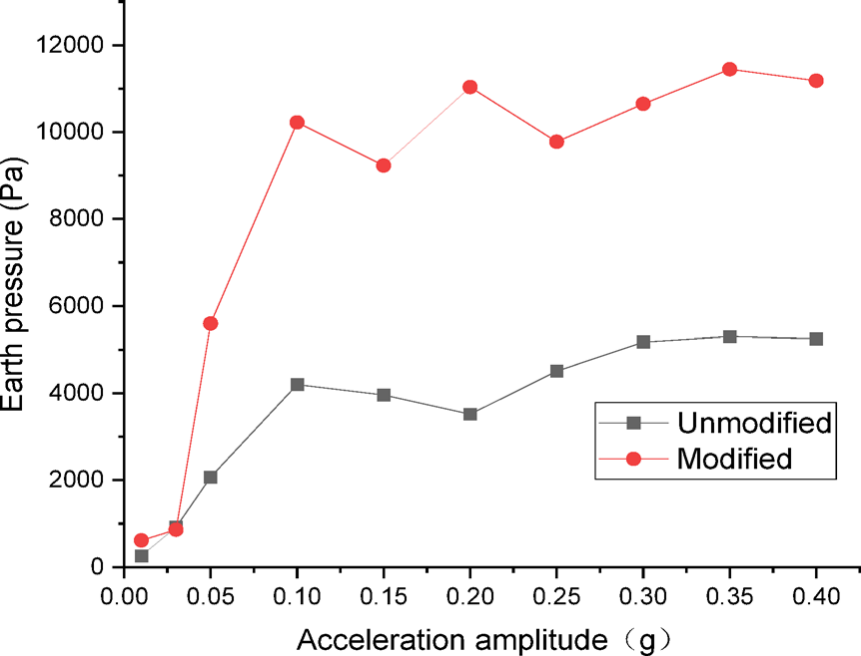
Earth pressure under Chi_Chi wave excitation at a wetting degree of 40%.

The data from dynamic earth pressure transducers were processed and plotted as curves. Figures 22 and 23 show the curves of peak dynamic earth pressure versus acceleration amplitude for the embankment under Chi_Chi wave excitation. As indicated in Fig. 22 for the wetting degree of 0%, the maximum dynamic earth pressure of the modified lateritic soil embankment is 18,228 Pa, which is 1.56 times that of the unmodified embankment at 11,659 Pa. As indicated in Fig. 23 for the wetting degree of 40%, the maximum dynamic earth pressure of the modified lateritic soil embankment is 11,446 Pa, which is 2.16 times that of the unmodified embankment at 5302 Pa.

**Fig. 24:**
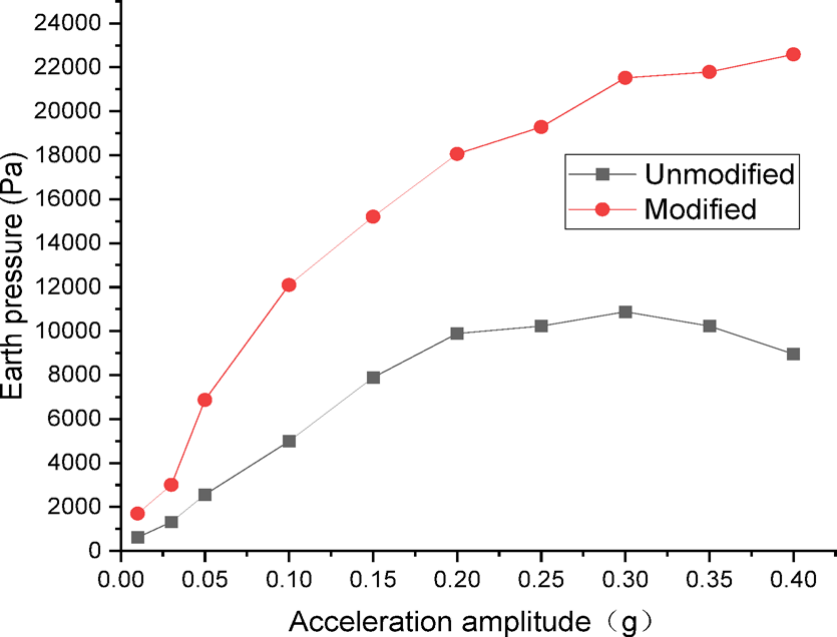
Earth pressure under NCALIF wave excitation at a wetting degree of 0%.

**Fig. 25:**
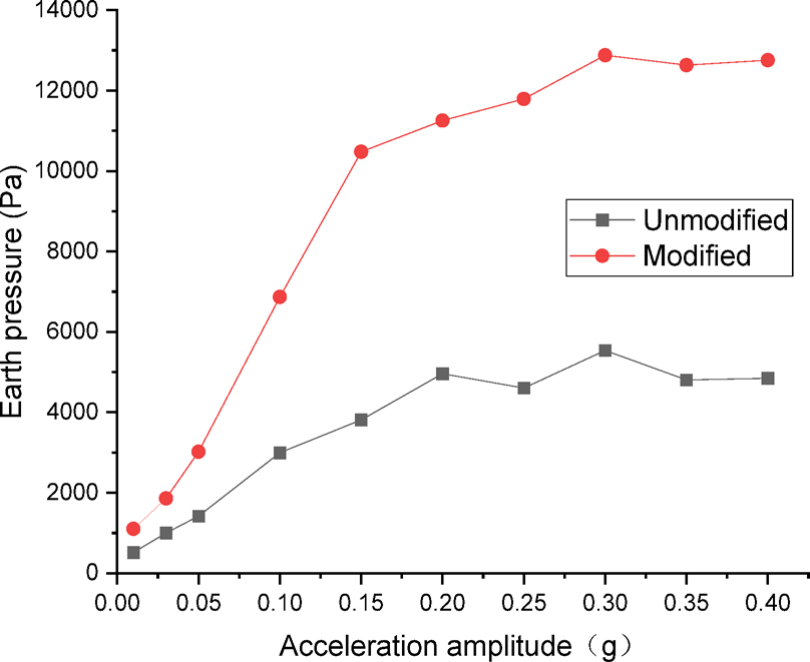
Earth pressure under NCALIF wave excitation at a wetting degree of 40%.

Figures 24 and 25 show the curves of peak dynamic earth pressure versus acceleration amplitude for the embankment under NCALIF wave excitation. As indicated in Fig. 24 for the wetting degree of 0%, the maximum dynamic earth pressure of the modified lateritic soil embankment is 22,589 Pa, which is 2.08 times that of the unmodified embankment at 10,877 Pa. As indicated in Fig. 25 for the wetting degree of 40%, the maximum dynamic earth pressure of the modified lateritic soil embankment is 12,878 Pa, which is 2.32 times that of the unmodified embankment at 5540 Pa.

**Fig. 26:**
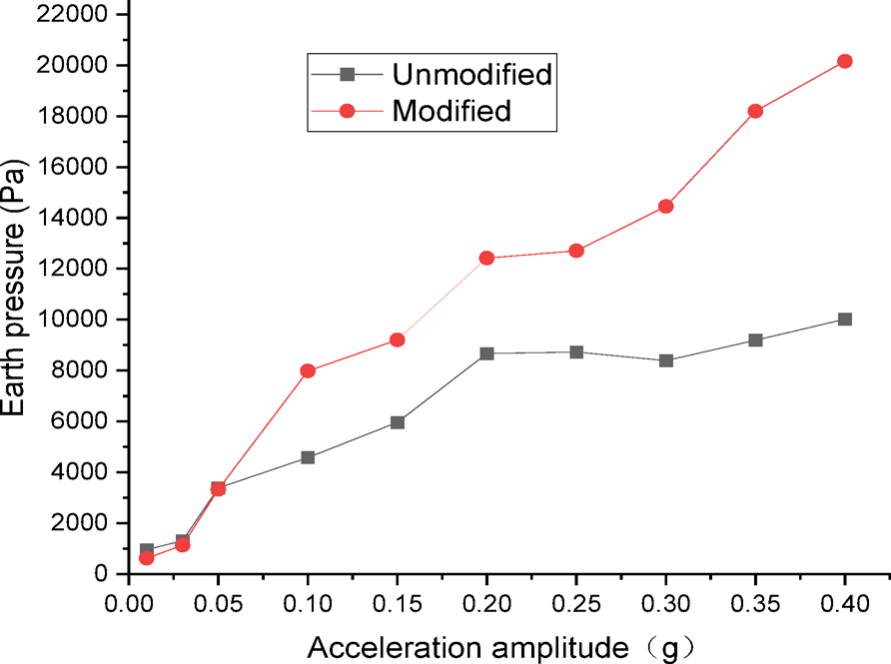
Earth pressure under SFERN wave excitation at a wetting degree of 0%.

**Fig. 27:**
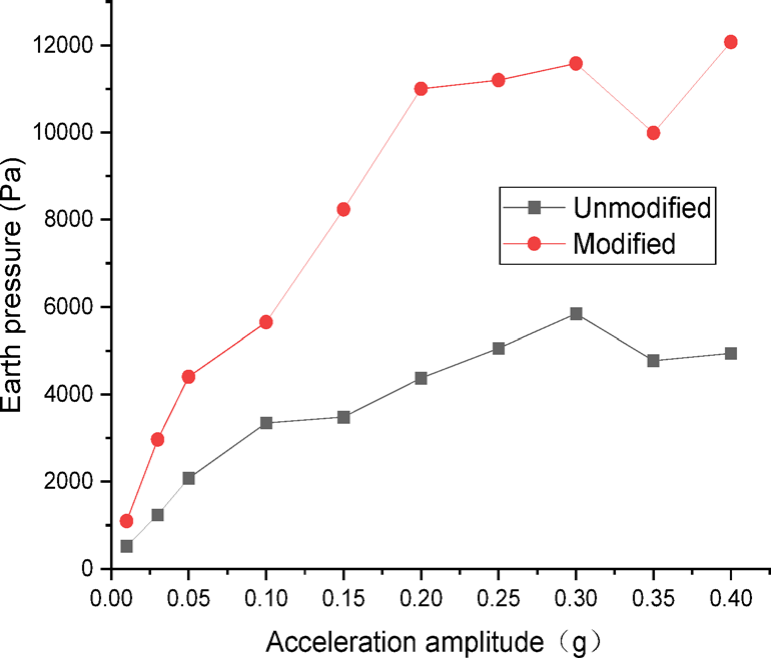
Earth pressure under SFERN wave excitation at a wetting degree of 40%.

Figures 26 and 27 show the curves of peak dynamic earth pressure versus acceleration amplitude for the embankment under SFERN wave excitation. As indicated in Fig. 26 for the wetting degree of 0%, the maximum dynamic earth pressure of the modified lateritic soil embankment is 20,159 Pa, which is 2.01 times that of the unmodified embankment at 10,023 Pa. As indicated in Fig. 27 for the wetting degree of 40%, the maximum dynamic earth pressure of the modified lateritic soil embankment is 12,078 Pa, which is 2.07 times that of the unmodified embankment at 5846 Pa.

Analysis of the dynamic earth pressure curves under the action of three types of seismic waves indicates that, for both unmodified and modified lateritic soil embankments, when the acceleration amplitude is less than 0.30 *g*, the earth pressure generally increases with the increase of acceleration amplitude both in the state of no wetting (wetting degree of 0%) and high wetting degree (wetting degree of 40%). Comparison of the two types of embankment under the same seismic wave, the same acceleration amplitude, and the same wetting degree indicates that the modification of lateritic soil enhances the stress transfer in the embankment soil. As a result, the peak dynamic earth pressure of the modified lateritic soil embankment is significantly higher than that of the unmodified embankment. Furthermore, comparison of the embankments under different wetting degrees but the same seismic wave and same acceleration amplitude indicates that wetting obviously weakens the stress transfer in the embankment soil. The dynamic earth pressure of the embankment at a wetting degree of 40% is significantly lower than that of the embankment with no wetting (wetting degree of 0%). Under the same working conditions, the peak dynamic earth pressure of the modified embankment is significantly higher than that of the unmodified embankment. Analysis suggests that the increased stiffness and enhanced permeability, which allows for rapid dissipation of dynamic pore water pressure, both contribute to improved stress transfer and thus increased shear strength.

## Conclusion

Model tests were conducted to study the dynamic response characteristics of embankments under combined action of wetting and vibration. Typical high-liquid-limit lateritic soils from central-southern China were used to build two scaled-down physical models of embankments: one model was built with unmodified lateritic soils and the other one with lateritic soils modified with lime at a content of 8%. The models were subjected to wetting of different degrees via simulated rainfall and were excited with white noise and three types of seismic waves: Chi_Chi, NCALIF, and SFERN. The following main conclusions are drawn:

1. There are significant differences between the dynamic properties of the two types of embankments. As the wetting degree of the embankment increases from 0 to 48%, the natural frequency of the modified lateritic soil embankment decreases from 29.77 Hz to 26.04 Hz, while the damping ratio increases from 0.1894 to 0.2438; for the unmodified lateritic soil embankment, the natural frequency decreases from 24.92 Hz to 23.76 Hz, and the damping ratio increases from 0.088 to 0.1367. At the same wetting degree, the natural frequency of the modified lateritic soil embankment is greater than that of the unmodified embankment, and the damping ratio of the modified embankment is significantly less than that of the unmodified embankment.
2. The PGA amplification effect of the two types of embankments under the action of three types of seismic waves was quantitatively analyzed. At the same wetting degree, the maximum PGA amplification factor of the modified embankment is always lower than that of the unmodified embankment. This indicates that modifying the lateritic soil for embankment construction reduces the PGA amplification effect of the embankment, thereby providing better performance in seismic mitigation. The maximum PGA amplification factors for both types of embankments predominantly occur within the low range of acceleration amplitude of no more than 0.20 *g*.
3. Quantitative analysis of peak dynamic pore water pressure under the action of three types of seismic waves indicates that, whether in the state of low wetting degree of 10% or high wetting degree of 40%, the peak dynamic pore water pressure of the modified lateritic soil embankment is significantly lower than that of the unmodified embankment. This indicates that the modification of lateritic soil with lime greatly improves the soil’s permeability, resulting in a significant dissipation effect of the pore water pressure.
4. Quantitative analysis of dynamic earth pressure under the action of three types of seismic waves indicates that, for both unmodified and modified lateritic soil embankments, the earth pressure increases with the increase of acceleration amplitude. Modification of lateritic soil enhances the stress transfer in the embankment soil. Under the same conditions, the peak dynamic earth pressure of the modified lateritic soil embankment is significantly greater than that of the unmodified embankment. For both types of embankments, wetting has an obvious weakening effect on stress transfer in the embankment soil. Under the same seismic wave and acceleration amplitude, the dynamic earth pressure of the embankment at a wetting degree of 40% is significantly lower than that of the embankment at no wetting (wetting degree of 0%).

## Data Availability

Data generated or analyzed during this study are available from the corresponding author upon reasonable request.
